# Influence of *PCDH9* (rs9540720) and narcissistic personality traits on the incidence of major depressive disorder in Chinese first-year university students: findings from a 2-year cohort study

**DOI:** 10.3389/fgene.2023.1267972

**Published:** 2024-02-07

**Authors:** Ruixue Xu, Zhaorui Liu, Hanyun Li, Linlin Luo, Yi Zheng, Fuqin Mu, Yujia Liu, Weixin Zhang, Ying Zhang, Jianli Wang, Yan Liu

**Affiliations:** ^1^ School of Public Health, Binzhou Medical University, Yantai, China; ^2^ School of Public Health, Jining Medical University, Jining, China; ^3^ NHC Key Laboratory of Mental Health (Peking University), National Clinical Research Center for Mental Disorders (Peking University Sixth Hospital), Peking University Sixth Hospital, Peking University Institute of Mental Health, Beijing, China; ^4^ Cheeloo College of Medicine, Shandong University, Jinan, China; ^5^ Department of Hematology, Tai’an City Central Hospital of Qingdao University, Tai’an, China; ^6^ School of Mental Health, Jining Medical University, Jining, China; ^7^ Melbourne School of Psychological Sciences, The University of Melbourne, Melbourne, VIC, Australia; ^8^ School of Clinical Medicine, Jining Medical University, Jining, China; ^9^ School of Public Health, University of Sydney, Sydney, NSW, Australia; ^10^ Department of Community Health and Epidemiology, Faculty of Medicine, Dalhousie University, Halifax, NS, Canada

**Keywords:** protocadherin gene, narcissistic personality traits, major depressive disorder, first-year university students, cohort study

## Abstract

**Objective:** The objective of this study was to explore the influence of the polymorphism of the protocadherin 9 (*PCDH9*) gene and the narcissistic personality trait (NPT) on the risk of major depressive disorder (MDD) in Chinese first-year university students.

**Methods:** A 2-year cohort study was conducted among Chinese first-year university students who were enrolled in 2018 from two universities in Shandong Province, China. The snapshot technique was used to detect the genotypes of *PCDH9* (rs9540720). The Chinese version of the Composite International Diagnostic Interview was used for the MDD assessment. The NPTs were measured by 11 items based on DSM-IV. Patient Health Questionnaire-9 and the Beck Anxiety Inventory were used to assess depressive and anxiety symptoms, respectively. Logistic regression modeling was carried out to examine the relationship between rs9540720, NPTs, and the incidence of MDD.

**Results:** A total of 5,327 students participated in the baseline and follow-up studies and provided their blood samples. *PCDH9* (rs9540720) (OR_GG+GA_ = 2.33, 95% CI: 1.35–4.02) and NPTs (OR_5–9_ = 2.26, 95% CI: 1.40–3.64) increased the risk of MDD onset. There was no multiplicative interaction between NPTs and Rs9540720 (OR = 1.51, 95% CI: 0.30–7.63). Furthermore, there was no additive interaction between them (RERI = 2.40, 95% CI: –0.82–5.62; AP = 0.47, 95% CI: –0.04–0.97; and S = 2.37, 95% CI: 0.54–10.33).

**Conclusion:** *PCDH9* (rs9540720) and more NPTs are the risk factors for the incidence of MDD in Chinese first-year university students.

## 1 Introduction

Major depressive disorder (MDD) is a major contributor to disability in the world (Filatova et al., 2021), with an estimated lifetime risk of 20% (Lin et al., 2022). Previous studies reported that the prevalence of MDD in college students was higher (5.9%) than that in the general population (3.4%) (Auerbach et al., 2016; Huang et al., 2019) and seemed to increase gradually (Acharya et al., 2018; Wang et al., 2022). First-year university students are in their late adolescence and early adulthood, and their physical and mental development is uncoordinated and unstable, with immature cognitive, emotional, and behavioral development (Wang, 2021). Furthermore, they are at a critical juncture burdened by family expectations and peer pressure (Wang, 2021). If college students cannot respond well to external stress, they can develop depressive moods, even MDD. MDD is the result of the interaction among demographic, biological, clinical, genetic, and environmental factors (Brown et al., 2013). Therefore, studying the impact of genetic and environmental factors on MDD in college students holds significant social value in preventing, intervening in, and improving the occurrence, development, and outcomes of MDD in this population.

When discussing the genetic factors that may contribute to a susceptibility to MDD, previous studies have focused on serotonin-related genes (Fakhoury et al., 2016) and brain-derived neurotrophic factor (*BDNF*) genes (Zelada et al., 2023). However, there has been limited evidence about the relationship between the protocadherin gene (*PCDH*) and MDD (Chang et al., 2018; Xiao et al., 2018). PCDH is a prominent family within the cadherin superfamily, predominantly expressed in the nervous system, playing a specific role as receptors in synaptic connection and signal transduction (Anitha et al., 2013). The PCDH family is further categorized into clustered PCDHs (cPCDHs) and non-clustered PCDHs (ncPCDHs) (Pancho et al., 2022). *PCDH9*, a member of the non-clustered PCDHs, is located on the human chromosome 13q21.32, with a length of approximately 927,503 nucleotides. Single-nucleotide polymorphism (SNP) data analysis is a high-throughput genotyping technique with significant research value in elucidating the genetic factors underlying diseases and in the diagnosis and treatment of diseases (Li et al., 2015). Xiao et al. (2018) have indicated an SNP in the *PCDH9* gene, rs9540720, which is significantly associated with MDD at the genome-wide scale.

Previous research has identified the association between personality disorders (PDs) and MDD (Fjermestad-Noll et al., 2019). Most studies on PDs focus on borderline personality and neurotic personality disorders, while less attention is paid to narcissistic personality disorder (NPD) (Caligor et al., 2015; Fjermestad-Noll et al., 2019; Wolf et al., 2023; Xia et al., 2023). Notably, NPD patients are among the most damaged and troublesome in the emergency departments and inpatient wards (Caligor et al., 2015). Research has shown that NPD is highly comorbid with other disorders and is associated with significant functional and psychological impairment (Miller et al., 2007; Stinson et al., 2008). The prevalence of NPD in the general population is estimated to be 0%–5.3% (Caligor et al., 2015). NPD is characterized by grandiose fantasies, a need for admiration, a sense of entitlement, and a lack of empathy (Caligor et al., 2015). Furthermore, the core psychological features of the disorder include a fragile sense of self-esteem, feelings of inferiority or emptiness, and emotional distress (Caligor et al., 2015). Meta-analysis results indicated that the comorbidity of MDD and PDs had worse outcomes and a poor prognosis than MDD alone (Newton-Howes et al., 2006). Additionally, a survey of PD comorbidities in people with early-onset and late-onset major depression revealed that NPD was more likely to occur in those with early-onset major depression (Fava et al., 1996). There are few studies on this topic on university/college students, so this study analyzed the effect of narcissistic personality traits (NPTs) on the incidence of MDD among college students.

In conclusion, the objectives of this study were to 1) examine the influence of the *PCDH9* gene and NPTs on the incidence of MDD among first-year university students and 2) to investigate the interactive effects of *PCDH9* gene polymorphism and NPTs on MDD.

## 2 Methods

### 2.1 Study population

This was a cohort study following first-year university students for 2 years. These students were enlisted from the Weifang Medical University and the Jining Medical University (Jining and Rizhao campuses) in Shandong Province, China. Compared with other provinces in China, Shandong Province is at the upper-middle level regarding economic development and the social and economic status (Liu et al., 2021). The students came from 25 provinces and autonomous regions. The study was approved by the Medical Ethics Committee of the Jining Medical University, and all participants submitted their informed consent before partaking in the study.

### 2.2 Data collection

In 2018, first-year students (9,928) from these universities were invited to participate in this study. Among them, 8,079 students (81.4%) provided their baseline data, and 5,687 students (57.28%) had their blood collected between April 2018 and October 2018. A 2-year follow-up study was conducted on 5,327 students (93.67%) who had never experienced MDD in their lifetime and who provided their blood samples. Out of these, 4,933 students (92.60%) completed the 1-year follow-up survey from April 2019 to October 2019. Due to the COVID-19 pandemic, a second follow-up survey was conducted from September to October 2020, and 3,312 students (62.17%) completed the study. These data were collected from the library using a computer-aided investigation system with logical checks and skips. To ensure student participation, a uniform time allocation was implemented for each class during the survey. Six well-trained investigators took charge of managing the sites and answering any questions raised by the participants. After the participants filled in and submitted all of their answers, all data were uploaded onto the local server of the Jining Medical University.

### 2.3 Measurements

#### 2.3.1 Major depressive disorder

Based on the DSM-IV criteria, the Chinese version of the Composite International Diagnostic Interview version 3.0 (CIDI-3.0) was used to measure MDD (American Psychiatric Association, 2013; Huang et al., 2010). CIDI-3.0 was a complete clinical diagnostics of structured interviews that well-trained non-professional interviewers could use to assess MDD in population-based mental health surveys. This study assessed lifetime MDD at the baseline and 12-month MDD at the 1-year and 2-year follow-up surveys to study new-onset MDD within 2 years. MDD was defined as conforming to the diagnostic criteria for MDD but not being evaluated for a history of mania or hypomania.

#### 2.3.2 SNP selection and genotyping

After obtaining informed consent from the participants, 5 mL of morning fasting vein whole-blood samples were collected. The whole-blood genomic DNA was extracted using the QIAamp 96 DNA QIAcube HT kit. Genotyping was accomplished via the second-generation sequencing technology Sequenom MassARRAY SNP (Gao et al., 2022), and a detailed testing report was provided by the testing company. The genotypes of rs9540720 were AA, GA, and GG. Among them, AA is the wild-type genotype, while GA and GG are mutant genotypes. Data are freely available for download at https://figshare.com/.

#### 2.3.3 Narcissistic personality trait

The 11-item questionnaire about the existence of the nine narcissistic traits, outlined in the DSM-IV (American Psychiatric Association, 2013), was used to measure NPTs at the baseline. The nine domain traits include self-importance, fantasies, uniqueness, a need for admiration, a sense of entitlement, exploitation, a lack of empathy, envy, and arrogance. The only domain trait described by the three items is “showing arrogance and haughty behaviors or attitudes,” which may be difficult to capture using a self-reporting format (Chinnarasri et al., 2021). The participants needed to answer if they were introduced by the wording, such as “In daily life, I always have a grandiose sense of self-importance.”, “In daily life, I am preoccupied with fantasies of unlimited success, power, brilliance, beauty, or ideal love.”, and “In daily life, I require excessive admiration.” Based on the DSM-IV criteria, the number of NPTs is classified into two levels: 0–4 and 5–9. The Cronbach’s α-value of this study was 0.75.

#### 2.3.4 Depressive symptoms

The depressive symptoms obtained via using Patient Health Questionnaire-9 (PHQ-9) were evaluated at the baseline. The PHQ-9 embodied nine items to assess depressive symptoms in the past 2 weeks. Every item is rated on the Likert type of four-point score: (0) “None,” (1) “Mild,” (2) “Moderate,” and (3) “Severe.” The total score of the PHQ-9 was 0–27, with higher scores illustrating graver depressive symptoms. The severity of depression is categorized as 0–9 (none or mild depression) and 10–27 (moderate or severe depression) (Liu and Wang, 2015). The Cronbach’s α of the Chinese version of the PHQ-9 among college students was 0.85 (Zhang et al., 2013). At the same time, this value in this study was 0.83.

#### 2.3.5 Anxiety symptoms

The anxiety symptoms assessed using the Beck Anxiety Inventory (BAI) were evaluated at the baseline. The BAI embodied 21 items to assess the anxiety symptoms in the past week. Every item is rated on the Likert type of four-point score: (1) “None,” (2) “Mild,” (3) “Moderate,” and (4) “Severe.” The total score of the BAI is 21–84, with higher scores illustrating graver anxiety symptoms. The total scores of 21–44 and 45–84 indicate none or mild anxiety symptoms and moderate or severe anxiety symptoms, respectively. The Cronbach’s α of the Chinese version of the BAI was 0.95 (Cheng et al., 2002), while that in this study was 0.93.

#### 2.3.6 Stressful life events

The Chinese version of the Adolescent Self-Rating Life Events Checklist (ASLEC) (Xin and Yao, 2015), a twenty-six-item scale developed to assess stressful life events in the past year, was used. The participants must answer “yes” or “no” to each item. The higher the total score, the more stressful life events one has experienced. The total score of ASLEC is 0–26. The number of stressful life events is classified into four levels: 0–3, 4–6, 7–9, and over 10. In this study, Cronbach’s α value was 0.81.

#### 2.3.7 Socio-demographic characteristics

Socio-demographic characteristics, such as age, sex, campus sites, major, single child, and residence, were also collected using a pre-designed questionnaire. For the exact questions, such as “What’s your age,” the participants need to fill in according to their actual situation; “What’s your sex,” which has two options “male” or “female”; “What’s your campus site,” which has the following options “Jining,” “Rizhao,” and “Weifang.” The participants must respond to each question accurately.

### 2.4 Statistical analysis

The genotype distribution of *PCDH9* (rs9540720) was tested using the Hardy–Weinberg equilibrium (HWE) test (Wittke-Thompson et al., 2005). Then, we compared the demographic characteristics under the classification of NPTs and *PCDH9* genotypes using independent samples for the *t*-test and chi-squared test for group differences.

The correlation between *PCDH9* (rs9540720), NPTs, and the risk of MDD was examined with the univariate logistic regression model. To determine MDD by NPTs and *PCDH9* (rs9540720), these variables (age, sex, family residence, single child, campus, PHQ-9 score, BAI score, and stressful life events) were included in the multivariate model as covariates. Additionally, the interaction between *PCDH9* (rs9540720) and NPT on the incidence of MDD was examined using both multiplicative and additive models. Finally, to analyze the combined effects of the gene and NPTs, a multivariable logistic regression analysis was carried out. The selection of the covariate variables for multivariable logistic regression was based on the results of the univariate analysis, professional knowledge, and clinical practice. The multivariable logistic regression analysis included a total of 11 variables, namely, sex, age, family residence, single child, major, campus, PHQ-9 score, BAI score, stressful life events, NPT, and rs9540720. In this statistical analysis, *p* <0.05 was the level of significance.

In this study, the formula used to calculate the required sample size is $n=\frac{{\left(Z_{1-\alpha /2}\sqrt{2\bar{\text{Pq}}}+Z_{\beta }\sqrt{P_{0}q_{0}+P_{1}q_{1}}\right)}^{2}}{{\left(P_{1}-P_{0}\right)}^{2}}$ . Based on the research conducted by Ferrari et al. (2013) and Ebert et al. (2019), the incidence of MDD in the general population was 3.00% and was 6.90% in college students. By entering these values into the formula, the required sample size was calculated to be 649. By taking attrition (20%) into consideration, the final estimated required minimum sample size was approximately 812. The sample size of this study was sufficient to achieve the required power.

## 3 Results

### 3.1 Demographic characteristics of the participants

The sample comprised 2,091 (39.35%) male students and 3,223 (60.65%) female students, with an average age of 18.39 ± 0.837. Most participants came from rural areas (64.21%). Nearly half of the participants attended the Jining Campus of the Jining Medical University, and 66.05% were majoring in medicine. Only 6.04% of students reported PHQ-9 scores of 10–27, while only 2.06% reported BAI scores of 45–84.

Table 1 displays the demographic characteristics of the participants under the different NPT subgroups. The number of participants scoring 5–9 on the NPT was 312, and the average age of these participants was 18.40 ± 0.837 (*χ* ^*2*^ = 18.10, *p* <0.001). As can be seen in Table 1, men (*χ* ^*2*^ = 2.14, *p* = 0.144), participants from urban areas (*χ* ^*2*^ = 11.07, *p* = 0.001), those who are a single child (*χ* ^*2*^ = 6.09, *p* = 0.014), those attending the Weifang or Rizhao campus (*χ* ^*2*^ = 8.17, *p* = 0.017), those with PHQ-9 scores of 10–27 (*χ* ^*2*^ = 246.91, *p* <0.001), BAI scores of 45–84 (*χ* ^*2*^ = 72.67, *p* <0.001), and those who have experienced more stressful life events (*χ* ^*2*^ = 119.55; *p* <0.001), tend to have a higher prevalence of NPTs. Age, sex, family residence, single child, major, campus, PHQ-9 score, BAI score, and stressful life events did not significantly differ between the genotype groups (*p* >0.05) (Supplementary Table S2). Age, sex, family residence, single child, campus, stressful life events, and rs9540720 were significantly different between follow-up non-completers and completers (*p* <0.05) (Supplementary Table S3).

**TABLE 1:** Demographic characteristics of 5,327 freshmen under the different NPT subgroups.

Variable	Category	NPT		*χ* ^*2*^/t	*p*
		0–4 *n* (%)	5–9 *n* (%)		
Age	Mean ± SD	18.40 ± 0.837	18.24 ± 0.819	−3.83	<0.001
Sex	Male	1,956 (93.54)	135 (6.46)	2.14	0.144
	Female	3,046 (94.51)	177 (5.49)		
Family residence	Urban areas	1,763 (92.69)	139 (7.31)	11.07	0.001
	Rural areas	3,239 (94.93)	173 (5.07)		
Single child	No	3,100 (94.77)	171 (5.23)	6.09	0.014
	Yes	1,856 (93.13)	137 (6.87)		
Major	Non-medicine	1,685 (93.40)	119 (6.60)	2.60	0.107
	Medicine	3,317 (94.50)	193 (5.50)		
Campus	Jining	2,190 (95.18)	111 (4.82)	8.17	0.017
	Rizhao	823 (93.10)	61 (6.90)		
	Weifang	1,989 (93.42)	140 (6.58)		
PHQ-9 score	0–9	4,764 (95.41)	229 (4.59)	246.91	<0.001
	10–27	238 (74.14)	83 (25.86)		
BAI score	21–44	4,908 (94.57)	282 (5.43)	72.67	<0.001
	45–84	82 (75.23)	27 (24.77)		
Stressful life events	0–3	1,170 (97.10)	35 (2.90)	119.55	<0.001
	4–6	1,462 (96.25)	57 (3.75)		
	7–9	1,260 (94.88)	68 (5.12)		
	≥10	1,093 (87.93)	150 (12.07)		

### 3.2 SNPs

In this study, the frequencies of AA, GG, and GA genotypes of rs9540720 were 906 (16.90%), 1,853 (34.68%), and 2,568 (48.42%), respectively. The HWE test results (*χ* ^*2*^ = 0.10, *p* = 0.950) indicated that the surveyed population had achieved a genetic equilibrium, suggesting that the data from this population survey were credible (Supplementary Table S1). In the logistic regression analysis, the results showed that GG + GA genotypes at rs9540720 had a higher risk of MDD than the AA genotype (Table 2).

**TABLE 2:** Univariate logistic regression analysis of NPTs and the *PCDH9* (rs9540720) gene in the incidence of MDD within 2 years.

Variable	Category	OR (95% CI)	*p*
Age	Mean ± SD	0.90 (0.74, 1.11)	0.329
Sex	Male	Reference	0.953
	Female	1.01 (0.73, 1.39)	
Family residence	Urban areas	Reference	0.690
	Rural areas	1.07 (0.77, 1.48)	
Single child	No	Reference	0.944
	Yes	0.99 (0.72, 1.36)	
Major	Non-medicine	Reference	0.935
	Medicine	1.01 (0.73, 1.41)	
Campus	Jining	Reference	
	Rizhao	0.78 (0.45, 1.34)	0.36
	Weifang	1.17 (0.85, 1.63)	0.34
PHQ-9 score	0–9	Reference	<0.001
	10–27	4.11 (2.74, 6.15)	
BAI score	21–44	Reference	0.027
	45–84	2.47 (1.11, 5.50)	
Stressful life events	0–3	Reference	
	4–6	1.48 (0.84, 2.62)	0.197
	7–9	1.44 (0.80, 2.60)	0.226
	≥10	3.39 (1.99, 5.78)	<0.001
NPT	0–4	Reference	0.001
	5–9	2.26 (1.40, 3.64)	
rs9540720	AA	Reference	0.002
	GG + GA	2.33 (1.35, 4.02)	

### 3.3 Univariate logistic regression analysis results

NPTs (OR_5–9_ = 2.26, 95% CI: 1.40–3.64) and rs9540720 (OR_GG+GA_ = 2.33, 95% CI: 1.35–4.02) had a positive correlation with the incidence of MDD within 2 years (Table 2).

### 3.4 The interaction analysis results

**FIGURE 1:**
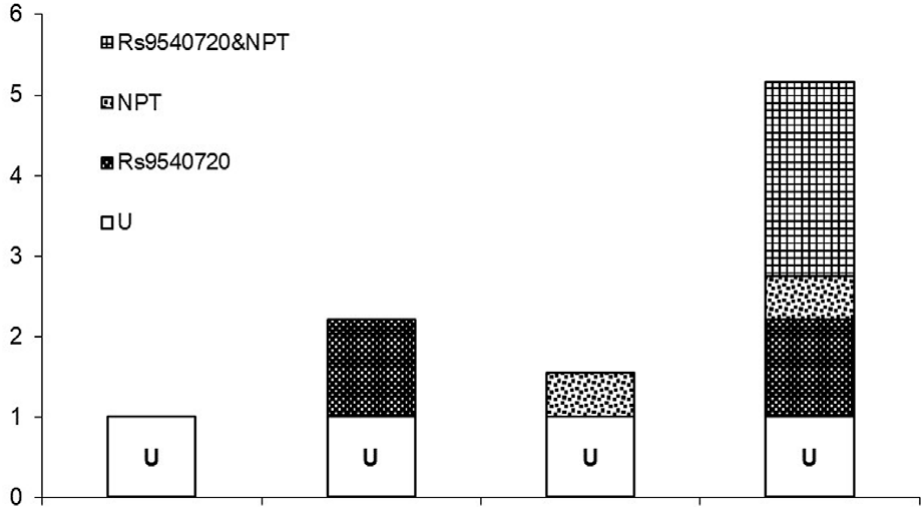
Relative risk with contributions from different marked exposure categories (U is a common reference category).

The multiplicative model included the interaction between NPTs and *PCDH9* (rs9540720) on the incidence of MDD. The result of NPTs × rs9540720 was not statistically significant (OR = 1.51, 95% CI: 0.30–7.63) (Table 3). Subsequently, both of them were further analyzed using an additive model. The additive model necessitated RERI ≠ 0, AP ≠ 0, and S ≠ 1. However, in this study, the 95% CI for the RERI index included 0, the 95% CI for the AP index included 0, and the 95% CI for the S index included 1, indicating no additive interaction (Table 4 and Figure 1).

**TABLE 3:** Interactions between rs9540720 (*PCDH9*) and NPTs in the multiplicative model.

Variable	*β*	OR (95% CI)	*p*
rs9540720	0.79	2.20 (1.23; 3.93)	0.008
NPT	0.44	1.56 (0.33; 7.43)	0.578
NPT × rs9540720	0.41	1.51 (0.30; 7.63)	0.620

**TABLE 4:** Interactions between rs9540720 (*PCDH9*) and NPTs in the additive model.

Variable	rs9540720	NPT	NPT & rs9540720
Regression coefficients	0.79	0.44	1.64
Cov rs9540720	0.09	0.08	0.08
Cov NPT	0.08	0.63	0.09
Cov rs9540720 and NPT	0.08	0.09	0.13
Exposure	RR	Lower	Upper
rs9540720	2.20	1.22	3.97
NPT	1.55	0.33	7.36
NPT & rs9540720	5.16	2.54	10.45
Measure	Estimate	Lower	Upper
RERI	2.40	−0.82	5.62
AP	0.47	−0.04	0.97
S	2.37	0.54	10.33

### 3.5 Multivariate logistic regression analysis results

The multivariate logistic regression model included age, sex, family residence, single child, major, campus, PHQ-9 score, BAI score, and stressful life events as control variables, and NPTs and rs9540720 as independent variables. The results showed that *PCDH9* (rs9540720) (OR_GG+GA_ = 2.33, 95% CI: 1.35–4.02) and NPTs (OR_5–9_ = 2.26; 95% CI: 1.40–3.64) were significantly associated with MDD in first-year university students (Table 5).

**TABLE 5:** Multivariate logistic regression analysis of NPTs and the *PCDH9* (rs9540720) gene in the incidence of MDD within 2 years.

Variable	Category	OR (95% CI)	*p*
Age	Mean ± SD	0.95 (0.77, 1.18)	0.654
Sex	Male	Reference	0.870
	Female	1.03 (0.73, 1.46)	
Family residence	Urban areas	Reference	0.306
	Rural areas	1.22 (0.84, 1.77)	
Single child	No	Reference	0.652
	Yes	1.09 (0.75, 1.59)	
Major	Non-medicine	Reference	0.980
	Medicine	1.01 (0.68, 1.49)	
Campus	Jining	Reference	
	Rizhao	0.54 (0.28, 1.06)	0.073
	Weifang	1.06 (0.73, 1.54)	0.747
PHQ-9 score	0–9	Reference	<0.001
	10–27	2.87 (1.81, 4.55)	
BAI score	21–44	Reference	0.814
	45–84	1.10 (0.47, 2.65)	
Stressful life events	0–3	Reference	
	4–6	1.37 (0.80, 2.36)	0.252
	7–9	1.34 (0.77, 2.34)	0.307
	≥10	2.54 (1.52, 4.25)	<0.001
NPT	0–4	Reference	0.001
	5–9	2.26 (1.40, 3.64)	
rs9540720	AA	Reference	0.002
	GG + GA	2.33 (1.35,4.02)	

## 4 Discussion

This cohort study investigated the effects of *PCDH9* (rs9540720) and NPTs on the incidence of MDD for over 2 years among first-year university students in China. The GG and GA genotypes of rs9540720 and NPTs were significantly associated with the risk of the new onset of MDD. There were no interactions between rs9540720 and NPTs on the incidence of MDD.

Consistent with Xiao et al. (2018), *PCDH9* (rs9540720) may be linked to MDD, with GG and GA genotype carriers showing a higher susceptibility to MDD. Kim et al. (2010) observed the significant role of non-clustered PCDHs in neuronal development, especially in the hippocampus and dentate gyrus. This type of PCDH also exhibited a region-dependent expression along the septotemporal axis during the early postnatal period and participated in the function of the hippocampal circuit (Kim et al., 2010). In addition, Hoshina et al. (2013) identified the region-dependent PCDH expression in other brain regions, including the basal ganglia. Extensive cortical and subcortical interconnections exist between the basal ganglia and frontal lobes (Bennett, 2011). The occurrence and development of MDD are closely related to the hippocampus, basal ganglia, and frontal lobe (Gunaydin and Kreitzer, 2016; Maller et al., 2018; Pizzagalli and Roberts, 2022). Considering this, non-clustered PCDHs are highly associated with MDD. Moreover, Bruining et al. (2015) found that *PCDH9* played a role in developing sensory cortex and sensorimotor phenotypes. Deficiencies in *PCDH9* can lead to specific long-term social and non-social (object) recognition disorders, which may affect an individual’s emotion regulation capacity and the capacity to reduce positive emotions. Given the fact that dysfunctional emotion regulation is a core characteristic of MDD (Rive et al., 2013), these findings underscore the potential relevance of *PCDH9* in the incidence of MDD.

This study showed that NPTs may increase the risk of MDD, supported by the physiological evidence that patients with NPD have a smaller volume of gray matter (GM) in the left anterior insula than that in the general population (Schulze et al., 2013). Wang et al. (2014) noted a positive correlation between the volume of GM in the left anterior insula and self-reported empathy. MDD is associated with empathy deficits, particularly in adults during earlier stages of life, along with that in children and adolescents (Hoffmann et al., 2016). Notably, children who experience stressful life events tend to emotionally manifest greater emotional egocentrism and deficits in emotional conflict processing while physiologically manifesting reduced functional coupling between the dorsolateral prefrontal cortex (DLPFC) and rSMG (Steinbeis et al., 2015; Biermann et al., 2022). Caligor et al. (2015) characterize the symptoms of NPD as having a fragile sense of self-esteem and a strong sense of emptiness. Individuals with NPTs may become more vulnerable when their intense need for self-admiration and idealistic expectations of themselves and others are unmet (Tritt et al., 2010). Increased vulnerability is closely related to the avoidance-oriented structure, predisposing individuals to neuroticism, anxiety, or depression (Tritt et al., 2010; Krizan and Herlache, 2018). NPD can be difficult to diagnose and may be overlooked. Therefore, it is crucial for individuals, especially students, to pay attention to the self-screening of NPTs while also being mindful of their mental health.

The study found no interaction between *PCDH9* (rs9540720) and NPTs in the incidence of MDD. This lack of interaction may be because the participants were only first-year university students who may be reluctant in displaying their narcissistic traits. Therefore, future research could investigate whether *PCDH9* (rs9540720) and NPTs interact in the incidence of MDD in a more diverse and representative sample.

This study had several limitations. First, data collection relied on a questionnaire, leading to a potential self-report and recall bias, which may have influenced the findings. Second, the screening for MDD, in this study, was based on CIDI-3.0 rather than on clinical diagnoses from psychiatrists.

Individual gene detection and personality trait screening might be valuable in the future clinical practice for MDD prevention. Future research on MDD in college students should be focused not only on students’ emotional changes but also on their personality traits and even on the molecular mechanisms of specific genes.

## Data Availability

The original contributions presented in the study are publicly available. These data can be found at: https://figshare.com/articles/dataset/dx_doi_org_10_6084_m9_figshare_24136164/24136164.

## References

[B1] Acharya L. Jin L. Collins W. (2018). College life is stressful today-emerging stressors and depressive symptoms in college students. J. Am. Coll. Health. 66, 655–664. 10.1080/07448481.2018.1451869 29565759

[B2] American Psychiatric Association (2013). Diagnostic and statistical manual of mental disorders. Washington, D.C.: American Psychiatric Association. 10.1038/nature21420

[B3] Anitha A. Thanseem I. Nakamura K. Yamada K. Iwayama Y. Toyota T. (2013). Protocadherin α (PCDHA) as a novel susceptibility gene for autism. J. Psychiatry. Neurosci. 38 (3), 192–198. 10.1503/jpn.120058 23031252 PMC3633712

[B4] Auerbach R. P. Alonso J. Axinn W. G. Cuijpers P. Ebert D. D. Green J. G. (2016). Mental disorders among college students in the world health organization world mental health surveys. Psychol. Med. 46 (14), 2955–2970. 10.1017/S0033291716001665 27484622 PMC5129654

[B48] Bennett M. R. (2011). The prefrontal-limbic network in depression: Modulation by hypothalamus, basal ganglia and midbrain. Prog. Neurobiol. 93 (4), 468–487. 10.1016/j.pneurobio.2011.01.006 21349315

[B5] Biermann L. Wunram H. L. Pokorny L. Breitinger E. Großheinrich N. Jarczok T. A. (2022). Changes in the TMS-evoked potential N100 in the dorsolateral prefrontal cortex as a function of depression severity in adolescents. J. Neural. Transm. (Vienna) 129 (11), 1339–1352. 10.1007/s00702-022-02539-9 36029418 PMC9550695

[B6] Brown G. W. Ban M. Craig T. K. Harris T. O. Herbert J. Uher R. (2013). Serotonin transporter length polymorphism, childhood maltreatment, and chronic depression: a specific gene-environment interaction. Depress. Anxiety. 30 (1), 5–13. 10.1002/da.21982 22847957

[B7] Bruining H. Matsui A. Oguro-Ando A. Kahn R. S. Van't Spijker H. M. Akkermans G. (2015). Genetic mapping in mice reveals the involvement of Pcdh9 in long-term social and object recognition and sensorimotor development. Biol. Psychiatry. 78 (7), 485–495. 10.1016/j.biopsych.2015.01.017 25802080

[B8] Caligor E. Levy K. N. Yeomans F. E. (2015). Narcissistic personality disorder: diagnostic and clinical challenges. Am. J. Psychiatry 172 (5), 415–422. 10.1176/appi.ajp.2014.14060723 25930131

[B9] Chang H. Hoshina N. Zhang C. Ma Y. Cao H. Wang Y. (2018). The protocadherin 17 gene affects cognition, personality, amygdala structure and function, synapse development and risk of major mood disorders. Mol. Psychiatry. 23 (2), 400–412. 10.1038/mp.2016.231 28070120 PMC5794872

[B10] Cheng S. K.-W. Wong C. W. Wong K. C. Chong G. S.-C. Wong M. T.-P. Chang S. S.-Y. (2002). A study of psychometric properties, normative scores, and factor structure of the Beck Anxiety Inventory–the Chinese version. [Chinese]. Chin. J. Clin. Psychol. 10 (1), 4–6. 10.16128/j.cnki.1005-3611.2002.01.002

[B11] Chinnarasri P. Wongpakaran N. Wongpakaran T. (2021). Developing and validating the narcissistic personality scale (NPS) among older Thai adults using rasch analysis. Healthc. (Basel) 9 (12), 1717. 10.3390/healthcare9121717 PMC8701268 34946443

[B12] Ebert D. D. Buntrock C. Mortier P. Auerbach R. Weisel K. K. Kessler R. C. (2019). Prediction of major depressive disorder onset in college students. Depress Anxiety 36 (4), 294–304. 10.1002/da.22867 30521136 PMC6519292

[B13] Fakhoury M. (2016). Revisiting the serotonin hypothesis: implications for major depressive disorders. Mol. Neurobiol. 53 (5), 2778–2786. 10.1007/s12035-015-9152-z 25823514

[B14] Fava M. Alpert J. E. Borus J. S. Nierenberg A. A. Pava J. A. Rosenbaum J. F. (1996). Patterns of personality disorder comorbidity in early-onset versus late-onset major depression. Am. J. Psychiatry. 153 (10), 1308–1312. 10.1176/ajp.153.10.1308 8831439

[B15] Ferrari A. J. Somerville A. J. Baxter A. J. Norman R. Patten S. B. Vos T. (2013). Global variation in the prevalence and incidence of major depressive disorder: a systematic review of the epidemiological literature. Psychol. Med. 43 (3), 471–481. 10.1017/S0033291712001511 22831756

[B16] Filatova E. V. Shadrina M. I. Slominsky P. A. (2021). Major depression: one brain, one disease, one set of intertwined processes. Cells 10 (6), 1283. 10.3390/cells10061283 34064233 PMC8224372

[B17] Fjermestad-Noll J. Ronningstam E. Bach B. Rosenbaum B. Simonsen E. (2019). Characterological depression in patients with narcissistic personality disorder. Nord. J. Psychiatry. 73 (8), 539–545. 10.1080/08039488.2019.1664630 31517547

[B18] Gao C. Luo L. L. Yue S. Wang F. T. Duan X. M. Qian Y. D. (2022). Gender differences of genetic etiology in the incidence of major depressive disorder among Han freshmen. Natl. Med. J. China. 102 (19), 1437–1444. 10.3760/112137-20220130-00224 35599408

[B19] Gunaydin L. A. Kreitzer A. C. (2016). Cortico-basal ganglia circuit function in psychiatric disease. Annu. Rev. Physiol. 78, 327–350. 10.1146/annurev-physiol-021115-105355 26667072

[B20] Hoffmann F. Banzhaf C. Kanske P. Gärtner M. Bermpohl F. Singer T. (2016). Empathy in depression: egocentric and altercentric biases and the role of alexithymia. J. Affect. Disord. 199, 23–29. 10.1016/j.jad.2016.03.007 27060429

[B21] Hoshina N. Tanimura A. Yamasaki M. Inoue T. Fukabori R. Kuroda T. (2013). Protocadherin 17 regulates presynaptic assembly in topographic corticobasal Ganglia circuits. Neuron 78 (5), 839–854. 10.1016/j.neuron.2013.03.031 23684785

[B50] Huang Y. Xie S. Lu J. Xu J. Dang W. Li Y. (2010). Community-based evaluation of the reliability and validity of Chinese version of composite international diagnostic Interview-3.0. Chin. Ment. Health. J. 24, 21–24+28. 10.3969/j.issn.1000-6729.2010.01.005

[B22] Huang Y. Wang Y. Wang H. Liu Z. Yu X. Yan J. (2019). Prevalence of mental disorders in China: a cross-sectional epidemiological study. Lancet Psychiatry 6 (3), 211–224. 10.1016/S2215-0366(18)30511-X 30792114

[B23] Kim S. Y. Mo J. W. Han S. Choi S. Y. Han S. B. Moon B. H. (2010). The expression of non-clustered protocadherins in adult rat hippocampal formation and the connecting brain regions. Neuroscience 170 (1), 189–199. 10.1016/j.neuroscience.2010.05.027 20541594

[B24] Krizan Z. Herlache A. D. (2018). The narcissism spectrum model: a synthetic view of narcissistic personality. Pers. Soc. Psychol. Rev. 22 (1), 3–31. 10.1177/1088868316685018 28132598

[B25] Li P. Guo M. Wang C. Liu X. Zou Q. (2015). An overview of SNP interactions in genome-wide association studies. Brief. Funct. Genomics. 14 (2), 143–155. 10.1093/bfgp/elu036 25241224

[B26] Lin H. C. Xirasagar S. Wang C. H. Cheng Y. F. Yang T. H. (2022). Increased risk of major depressive disorder following tinnitus: a population-based study. Front. Neurol. 13, 836842. 10.3389/fneur.2022.836842 35401414 PMC8992000

[B27] Liu Y. Li B. H. Hao F. C. Wang B. Zhu J. Liu D. B. (2021). Associations between borderline personality disorder features and the risk of first onset major depressive disorder: findings from a 2-year longitudinal study in a sample of first-year university students in China. J. Affect. Disord. 295, 5–10. 10.1016/j.jad.2021.08.008 34385011

[B28] Liu Y. Wang J. (2015). Validity of the patient health questionnaire-9 for DSM-IV major depressive disorder in a sample of Canadian working population. J. Affect. Disord. 187, 122–126. 10.1016/j.jad.2015.07.044 26331686

[B29] Maller J. J. Broadhouse K. Rush A. J. Gordon E. Koslow S. Grieve S. M. (2018). Increased hippocampal tail volume predicts depression status and remission to anti-depressant medications in major depression. Mol. Psychiatry. 23 (8), 1737–1744. 10.1038/mp.2017.224 29133948

[B30] Miller J. D. Campbell W. K. Pilkonis P. A. (2007). Narcissistic personality disorder: relations with distress and functional impairment. Compr. Psychiatry. 48 (2), 170–177. 10.1016/j.comppsych.2006.10.003 17292708 PMC1857317

[B31] Newton-Howes G. Tyrer P. Johnson T. (2006). Personality disorder and the outcome of depression: meta-analysis of published studies. Br. J. Psychiatry 188, 13–20. 10.1192/bjp.188.1.13 16388064

[B49] Pancho A. Mitsogiannis M. D. Aerts T. Dalla Vecchia M. Ebert L. K. Geenen L. (2022). Modifying PCDH19 levels affects cortical interneuron migration. Front. Neurosci. 16, 887478. 10.3389/fnins.2022.887478 36389226 PMC9642031

[B32] Pizzagalli D. A. Roberts A. C. (2022). Prefrontal cortex and depression. Neuropsychopharmacology 47 (1), 225–246. 10.1038/s41386-021-01101-7 34341498 PMC8617037

[B33] Rive M. M. van Rooijen G. Veltman D. J. Phillips M. L. Schene A. H. Ruhé H. G. (2013). Neural correlates of dysfunctional emotion regulation in major depressive disorder. A systematic review of neuroimaging studies. Neurosci. Biobehav. Rev. 37 (10 Pt 2), 2529–2553. 10.1016/j.neubiorev.2013.07.018 23928089

[B34] Schulze L. Dziobek I. Vater A. Heekeren H. R. Bajbouj M. Renneberg B. (2013). Gray matter abnormalities in patients with narcissistic personality disorder. J. Psychiatr. Res. 47 (10), 1363–1369. 10.1016/j.jpsychires.2013.05.017 23777939

[B35] Steinbeis N. Bernhardt B. C. Singer T. (2015). Age-related differences in function and structure of rSMG and reduced functional connectivity with DLPFC explains heightened emotional egocentricity bias in childhood. Soc. Cogn. Affect. Neurosci. 10 (2), 302–310. 10.1093/scan/nsu057 24771281 PMC4321629

[B36] Stinson F. S. Dawson D. A. Goldstein R. B. Chou S. P. Huang B. Smith S. M. (2008). Prevalence, correlates, disability, and comorbidity of DSM-IV narcissistic personality disorder: results from the wave 2 national epidemiologic survey on alcohol and related conditions. J. Clin. Psychiatry. 69 (7), 1033–1045. 10.4088/jcp.v69n0701 18557663 PMC2669224

[B37] Tritt S. M. Ryder A. G. Ring A. J. Pincus A. L. (2010). Pathological narcissism and the depressive temperament. J. Affect. Disord. 122 (3), 280–284. 10.1016/j.jad.2009.09.006 19800134

[B38] Wang Q. F. Zhang Z. W. Dong F. Chen L. G. Zheng L. Guo X. Y. (2014). Anterior insula GABA levels correlate with emotional aspects of empathy: a proton magnetic resonance spectroscopy study. PloS. One. 9 (11), e113845. 10.1371/journal.pone.0113845 25419976 PMC4242717

[B39] Wang S. Y. (2021). Implicit narcissism and depression in college students: explain to the mediation and intervention study. a master's degree thesis. Wuhan, China: central China normal university.

[B40] Wang Y. J. Liu C. J. Song J. G. Guo Z. J. Wang H. L. Wang C. S. (2022). An epidemiological survey of depression and anxiety disorders among people aged 18 years and above in Henan province in 2021. Chin. J. Clin. Psychol. 55, 129–137.

[B41] Wittke-Thompson J. K. Pluzhnikov A. Cox N. J. (2005). Rational inferences about departures from Hardy-Weinberg equilibrium. Am. J. Hum. Genet. 76, 967–986. 10.1086/430507 15834813 PMC1196455

[B42] Wolf J. Reinhard M. A. Goerigk S. Barton B. B. Burkhardt G. Tang J. (2023). Suicidal behaviors and adverse childhood experiences: a cross-diagnostic study in persistent depressive disorder and borderline personality disorder. Psychiatry. Res. 330, 115562. 10.1016/j.psychres.2023.115562 37918208

[B43] Xia D. Han X. Zeng Y. Wang J. Xu K. Zhang T. (2023). Disease trajectory of high neuroticism and the relevance to psychiatric disorders: a retro-prospective cohort study. Acta. Psychiatr. Scand. 10.1111/acps.13645 38057974

[B44] Xiao X. Zheng F. Chang H. Ma Y. Yao Y. G. Luo X. J. (2018). The gene encoding protocadherin 9 (PCDH9), a novel risk factor for major depressive disorder. Neuropsychopharmacology 43 (5), 1128–1137. 10.1038/npp.2017.241 28990594 PMC5854803

[B45] Xin X. Yao S. (2015). Validity and reliability of the adolescent self-rating life events checklist in middle school students. Chin. Ment. Health. J. 29, 355–360.

[B46] Zelada M. I. Garrido V. Liberona A. Jones N. Zúñiga K. Silva H. (2023). Brain-derived neurotrophic factor (BDNF) as a predictor of treatment response in major depressive disorder (MDD): a systematic review. Int. J. Mol. Sci. 24 (19), 14810. 10.3390/ijms241914810 37834258 PMC10572866

[B47] Zhang Y. L. Liang W. Chen Z. M. Zhang H. M. Zhang J. H. Weng X. Q. (2013). Validity and reliability of patient health Questionnaire-9 and patient health Questionnaire-2 to screen for depression among college students in China. Asia Pac Psychiatry 5, 268–275. 10.1111/appy.12103 24123859

