# Supplementary material for: Influence of PCDH9 (rs9540720) and narcissistic personality traits on the incidence of major depressive disorder in Chinese first-year university students: findings from a 2-year cohort study
Source: Front Genet. 2024 Feb 7;14:1267972. doi: 10.3389/fgene.2023.1267972 (PMC10879931; doi:10.3389/fgene.2023.1267972)
Supplement: Supplementary file 1 [file Table1.pdf]

Supplementary Table 1

Results of Hardy-Weinberg Equilibrium (HWE) test for rs9540720

SNP	Genotype	Observed Frequency N (%)	Expected Frequency N (%)	χ^2	*P*
Rs9540720					
	AA	906 (17.01)	901 (16.90)	0.10	0.950
	GG	1853 (34.79)	1847 (34.68)		
	GA	2568 (48.21)	2579 (48.42)		
