# Supplementary material for: Influence of PCDH9 (rs9540720) and narcissistic personality traits on the incidence of major depressive disorder in Chinese first-year university students: findings from a 2-year cohort study
Source: Front Genet. 2024 Feb 7;14:1267972. doi: 10.3389/fgene.2023.1267972 (PMC10879931; doi:10.3389/fgene.2023.1267972)
Supplement: Supplementary file 2 [file Table2.pdf]

Supplementary Table 2

The demographic characteristics of 5,327 freshmen with different genotypes of the rs9540720

Variables	Categories	Rs9540720		χ^2 /t	*P*
		AA n (%)	GG + GA n (%)		
Age	Mean ± SD	18.41 ± 0.860	18.39 ± 0.832	-0.44	0.663
Sex	Male	349 (16.64)	1748 (83.36)	0.33	0.568
	Female	557 (17.24)	2673 (82.76)		
Family residence	Urban areas	321 (16.85)	1584 (83.15)	0.05	0.820
	Rural areas	585 (17.10)	2837 (82.90)		
Single child	No	546 (16.65)	2734 (83.35)	0.54	0.464
	Yes	348 (17.43)	1649 (82.57)		
Major	Non-medicine	325 (17.98)	1483 (82.02)	1.82	0.178
	Medicine	581 (16.51)	2938 (83.49)		
Campus	Jining	402 (17.40)	1908 (82.60)	1.40	0.496
	Rizhao	139 (15.67)	748(84.33)		
	Weifang	365 (17.14)	1765(82.86)		
PHQ-9 score	0-9	853 (17.04)	4152 (82.96)	0.07	0.787
	10-27	53 (16.46)	269 (83.54)		
BAI score	21-44	883 (16.99)	4314 (83.01)	1.15	0.701
	45-84	17 (15.60)	92 (84.40)		
Stressful life events	0-3	203 (16.82)	1004(83.18)	2.23	0.526
	4-6	244 (16.04)	1277 (83.96)		
	7-9	241 (18.13)	1088 (81.87)		
	≥10	212 (17.03)	1033 (82.97)		
