# Supplementary material for: Influence of PCDH9 (rs9540720) and narcissistic personality traits on the incidence of major depressive disorder in Chinese first-year university students: findings from a 2-year cohort study
Source: Front Genet. 2024 Feb 7;14:1267972. doi: 10.3389/fgene.2023.1267972 (PMC10879931; doi:10.3389/fgene.2023.1267972)
Supplement: Supplementary file 3 [file Table3.pdf]

Supplementary Table 3

The demographic characteristics of follow-up non-completers and completers

Variables	Categories	Non-completers (%)	Completers (%)	χ^2 /t	*P*
Age	Mean ± SD	18.47 ± 0.948	18.34 ± 0.765	–4.21	<0.001
Sex	Male	864 (41.20)	1233 (58.80)	49.35	<0.001
	Female	1026 (31.77)	2203 (68.23)		
Family residence	Urban areas	711 (37.72)	1194 (62.68)	4.37	0.037
	Rural areas	1179 (34.46)	2242 (65.54)		
Single child	No	1127 (34.37)	2152 (65.63)	5.16	0.023
	Yes	748 (37.46)	1249 (62.54)		
Major	Non-medicine	673 (37.22)	1135 (62.78)	3.61	0.058
	Medicine	1217 (34.59)	2301 (65.41)		
Campus	Jining	882 (38.18)	1428 (61.82)	139.95	<0.001
	Rizhao	431 (48.65)	455 (51.35)		
	Weifang	577 (27.09)	1553 (72.91)		
PHQ-9 score	0-9	1788 (35.73)	3216 (64.27)	2.17	0.141
	10-27	102 (31.68)	220 (68.32)		
BAI score	21-44	1836 (35.33)	3360 (64.67)	2.20	0.138
	45-84	46 (42.20)	63 (57.80)		
Stressful life events	0-3	480 (39.77)	727 (60.23)	13.92	0.003
	4-6	511 (33.60)	1010 (66.40)		
	7-9	467 (35.17)	861 (64.83)		
	≥10	419 (33.65)	826 (66.35)		
NPT	0-4	1779 (35.57)	3222 (64.43)	0.21	0.647
	5-9	107 (34.29)	205 (65.71)		
Rs9540720	AA	295 (32.56)	611 (67.44)	4.08	0.043
	GG+GA	1595 (36.09)	2825 (63.91)		
