# Supplementary material for: Influence of PCDH9 (rs9540720) and narcissistic personality traits on the incidence of major depressive disorder in Chinese first-year university students: findings from a 2-year cohort study
Source: Front Genet. 2024 Feb 7;14:1267972. doi: 10.3389/fgene.2023.1267972 (PMC10879931; doi:10.3389/fgene.2023.1267972)
Supplement: Supplementary file 4 [file Table5.docx]

| **Table 5**  Multivariate logistic regression analysis of NPT and gene *PCDH9* (rs9540720) in the incidence of MDD within 2 years | | | |
| --- | --- | --- | --- |
| Variables | Categories | OR (95% CI) | *P* |
| Age | Mean ± SD | 0.95 (0.77, 1.18) | 0.654 |
| Sex | Male | Reference |  |
|  | Female | 1.03 (0.73 1.46) | 0.870 |
| Family residence | Urban areas | Reference |  |
|  | Rural areas | 1.22 (0.84, 1.77) | 0.306 |
| Single child | No | Reference |  |
|  | Yes | 1.09 (0.75, 1.59) | 0.652 |
| Major | Non-medicine | Reference |  |
|  | Medicine | 1.01 (0.68, 1.49) | 0.980 |
| Campus | Jining | Reference |  |
|  | Rizhao | 0.54 (0.28, 1.06) | 0.073 |
|  | Weifang | 1.06 (0.73, 1.54) | 0.747 |
| PHQ-9 score | 0-9 | Reference |  |
|  | 10-27 | 2.87 (1.81, 4.55) | <0.001 |
| BAI score | 21-44 | Reference |  |
|  | 45-84 | 1.10 (0.47, 2.65) | 0.814 |
| Stressful life events | 0-3 | Reference |  |
|  | 4-6 | 1.37 (0.80, 2.36) | 0.252 |
|  | 7-9 | 1.34 (0.77, 2.34) | 0.307 |
|  | ≥10 | 2.54 (1.52, 4.25) | <0.001 |
| NPT | 0-4 | Reference |  |
|  | 5-9 | 2.26 (1.40, 3.64) | 0.001 |
| Rs9540720 | AA | Reference |  |
|  | GG + GA | 2.33 (1.35,4.02) | 0.002 |
