# Supplementary material for: Influence of PCDH9 (rs9540720) and narcissistic personality traits on the incidence of major depressive disorder in Chinese first-year university students: findings from a 2-year cohort study
Source: Front Genet. 2024 Feb 7;14:1267972. doi: 10.3389/fgene.2023.1267972 (PMC10879931; doi:10.3389/fgene.2023.1267972)
Supplement: Supplementary file 5 [file Table4.docx]

| **Table 4**  Interactions between rs9540720 (*PCDH9*) and NPT in the additive model | | | |
| --- | --- | --- | --- |
| Variables | Rs9540720 | NPT | NPT & rs9540720 |
| Regr. coefficients | 0.79 | 0.44 | 1.64 |
| Cov rs9540720 | 0.09 | 0.08 | 0.08 |
| Cov NPT | 0.08 | 0.63 | 0.09 |
| Cov rs9540720 & NPT | 0.08 | 0.09 | 0.13 |
| Exposure | RR | Lower | Upper |
| Rs9540720 | 2.20 | 1.22 | 3.97 |
| NPT | 1.55 | 0.33 | 7.36 |
| NPT & Rs9540720 | 5.16 | 2.54 | 10.45 |
| Measure | Estimate | Lower | Upper |
| RERI | 2.40 | -0.82 | 5.62 |
| AP | 0.47 | -0.04 | 0.97 |
| S | 2.37 | 0.54 | 10.33 |
